# A synthesis and future research directions for tropical mountain ecosystem restoration

**DOI:** 10.1038/s41598-021-03205-y

**Published:** 2021-12-14

**Authors:** Tina Christmann, Imma Oliveras Menor

**Affiliations:** 1 School of Geography and the Environment, Environmental Change Institute, University of Oxford, South Parks Road, Oxford, OX1 3QY UK; 2 Worcester College, 1 Walton Street, Oxford, OX12HB UK

**Keywords:** Restoration ecology, Biodiversity, Tropical ecology

## Abstract

Many tropical mountain ecosystems (TME) are severely disturbed, requiring ecological restoration to recover biodiversity and ecosystem functions. However, the extent of restoration efforts across TMEs is not known due to the lack of syntheses on ecological restoration research. Here, based on a systematic review, we identify geographical and thematic research gaps, compare restoration interventions, and consolidate enabling factors and barriers of restoration success. We find that restoration research outside Latin-America, in non-forested ecosystems, and on socio-ecological questions is scarce. For most restoration interventions success is mixed and generally limited by dispersal and microhabitat conditions. Finally, we propose five directions for future research on tropical mountain restoration in the UN decade of restoration, ranging from scaling up restoration across mountain ranges, investigating restoration in mountain grasslands, to incorporating socio-economic and technological dimensions.

## Introduction

Tropical mountain ecosystems (TME) are hotspots of biodiversity^1,2^ and endemism^3^ and are located in tropical latitudes between 1000 and 4000 m asl, and the elevation gradients give rise to a variety of ecosystems including montane forests, montane cloud forests, forest-grassland treelines, mountain grasslands and azonal formations (Table 1). TME span across all continents in the tropical belt, and despite their small spatial extent of just over 4 million km^2^ (Table 1) they provide numerous ecosystem services to people and society, including carbon sequestration, water regulation and supply, timber and food provision, erosion control, and cultural services^4^.

**Table 1:** Definition and description of the five tropical mountain ecosystems, their ecological features and the total area in the tropics.

Ecosystem	Description	Examples of sub ecosystems	Elevation	Total area (km^2^) across the tropics
Mountain grasslands	Grass-dominated systems found above the treeline. Highly biodiverse with adaptations to strong abiotic stressors, such as tussock growth or conservative functional traits^18^	Alpine grasslands at high elevations (Andean *Puna* and *Páramos*), Montane grasslands at lower elevations (Western Ghats, *Campos Rupestres*)	*Páramos:* above 3000 m asl	~ 846,286 km^2^
			*Puna:* above 3400 m asl^18^	
			*Campos Rupestres:* 900-2000 m asl	Sum of all tropical-mountain grassland types extracted from^19^
Tree line ecotone	Transition ecosystem between tree life forms and graminoids, forbs or shrubs with a-seasonal growing patterns and controlled by temperature and/or land use^20^. Can assume different types of shapes, such as sharp transition, stunted trees, tree island outposts or gradual ecotone^21^	Shrublands, Sub-alpine Polylepis forest, High Andean Forest	Variable elevations, dependent on local topographical and climatological positions	No estimates available
Azonal formations	Spatially restricted non-zonal ecosystem that occur due to topographical or hydrological features	Mountain peatlands and bogs, Riparian ecosystems, Inselberg forests	Variable elevations	No estimates available
Montane cloud forest (hereafter ‘cloud forest’)	Forested ecosystem shaped by frequent fog immersion, wetness and windy conditions. Harbours a distinct tree and epiphyte community with functional adaptations to the mountain hydrology and high elevation conditions^22^	Lower montane cloud forest, Upper montane cloud forest, Sub-alpine cloud forest/’Elfin’ forest	Generally, between 1200–1500 m asl. But lower boundary of 400 masl on some islands—and of 2000 m asl on large mountain ranges^22^	~ 214,630 km^2^^22^ (i.e. 6% of all montane tropical forests)^22^
Montane forest	Elevation forest with colder temperatures and distinct abiotic conditions to lowland forests. Forests usually show higher stem density, lower DBH, stem length and leaf area index with increasing elevation^23^	Lower montane forest, upper montane forest, dry montane forest, wet montane forest, montane bamboo forest	500–3500 m asl	3,257,275 km^2^^22^
			Montane forests sensu* Strictu* 1000–3500 m asl	
			Pre-montane forests 500–1000 m asl	

Notwithstanding their tremendous biological importance and complexity, TME are still relatively understudied compared to temperate mountain systems^5^. In recent decades, TME have been experiencing increasing pressure from multiple external drivers and stressors, such as anthropogenic pressures due to agricultural encroachment, pasture conversion and population growth^6^, exotic plantations^7,8^, invasion by exotic animals^9^ and exotic plants^10–12^, as well as accelerating climate change impacts^13^. These drivers lead to severe degradation in TME, impacting all levels of ecological organization, such as disruption of ecosystem services, losses in community diversity, changes in species interactions, reductions of population sizes and lowered genetic diversity^14^. Degradation in TME is far-reaching and ubiquitous: Tovar et al.^15^ projected that climate change will alter 3–7% of tropical Andean biomes, resulting in a 31.4% loss in extent of high-altitudinal Páramo grasslands due to replacement by montane forests by 2039. Further, Helmer et al.^16^ indicate that in the next 25–45 years, reductions in cloud immersion are estimated to diminish 57–80% of Neotropical montane cloud forests. Hall et al.^17^ estimate that the Tanzanian Eastern Arc mountains have lost 25% of forested areas since 1955, with deforestation rates of 57% in sub-montane forests (800–1200 m).

At the same time, socio-economic drivers have led to migrations of people from tropical mountains to urban areas, abandoning many previously cultivated and inhabited areas^24–26^ and creating a large opportunity for ecosystem recovery and restoration across many TME.

Restoration of biodiverse ecosystems, such as TME, has the potential to simultaneously recover lost biodiversity and ecosystem functioning and improve local livelihoods^27^, and has recently come to the fore of global conservation efforts^28^. Restoration is defined as *“the process of assisting the recovery of an ecosystem that has been degraded, damaged or destroyed*”^29^ and, as such, encompasses a broad suite of approaches ranging from passive restoration, to assisted recovery and active restoration. The urgency for global restorative actions culminated in global restoration pledges like the 2011 Bonn Challenge and the proclamation of the UN Decade of Ecosystem Restoration. Motivations to restore damaged ecosystems include conserving biodiversity (specific habitats or species), enhancing ecosystem processes (such as nutrient cycling), combatting climate change (through carbon storage or adaptation), and providing ecosystem services (such as water regulation or food provision) for cultural and spiritual reasons^30^. While restoration is by no means a replacement for protection of intact ecosystems, it is a useful complementary conservation strategy to recover degraded land, such as in the case of reforestation^27^.

Thus far, tropical ecosystem restoration has pre-dominantly been focussed around the lowlands, where restoration ecology has been thoroughly studied and synthesized in recent years^31–34^ and various restoration methods have been tested and compared^34^. On the other hand, only a handful of reviews have addressed restoration questions related to TME, most of which are specific to a single tropical mountain ecosystem type or to a specific restoration context^31–33,35–39^.

To the best of our knowledge there is no pantropical synthesis of scientific knowledge on ecosystem restoration in tropical mountain ecosystems. By completing a systematic review of 980 search entries and a meta-analysis of 176 systematically selected TME restoration studies following the SALSA methodology^40^ (see “Methods”), we address four key questions: (1) When, where, how and why does tropical mountain restoration research take place? (2) What restoration methods are used in TME restoration? (3) What limits or promotes success in TME restoration? We then discuss these questions in the light of climate change in tropical mountains and present research directions for TME restoration research for the upcoming UN Decade of Restoration.

## The state of science in tropical mountain restoration

### Tropical mountain restoration research reveals strong geographical and research nodes

**Figure 1:**
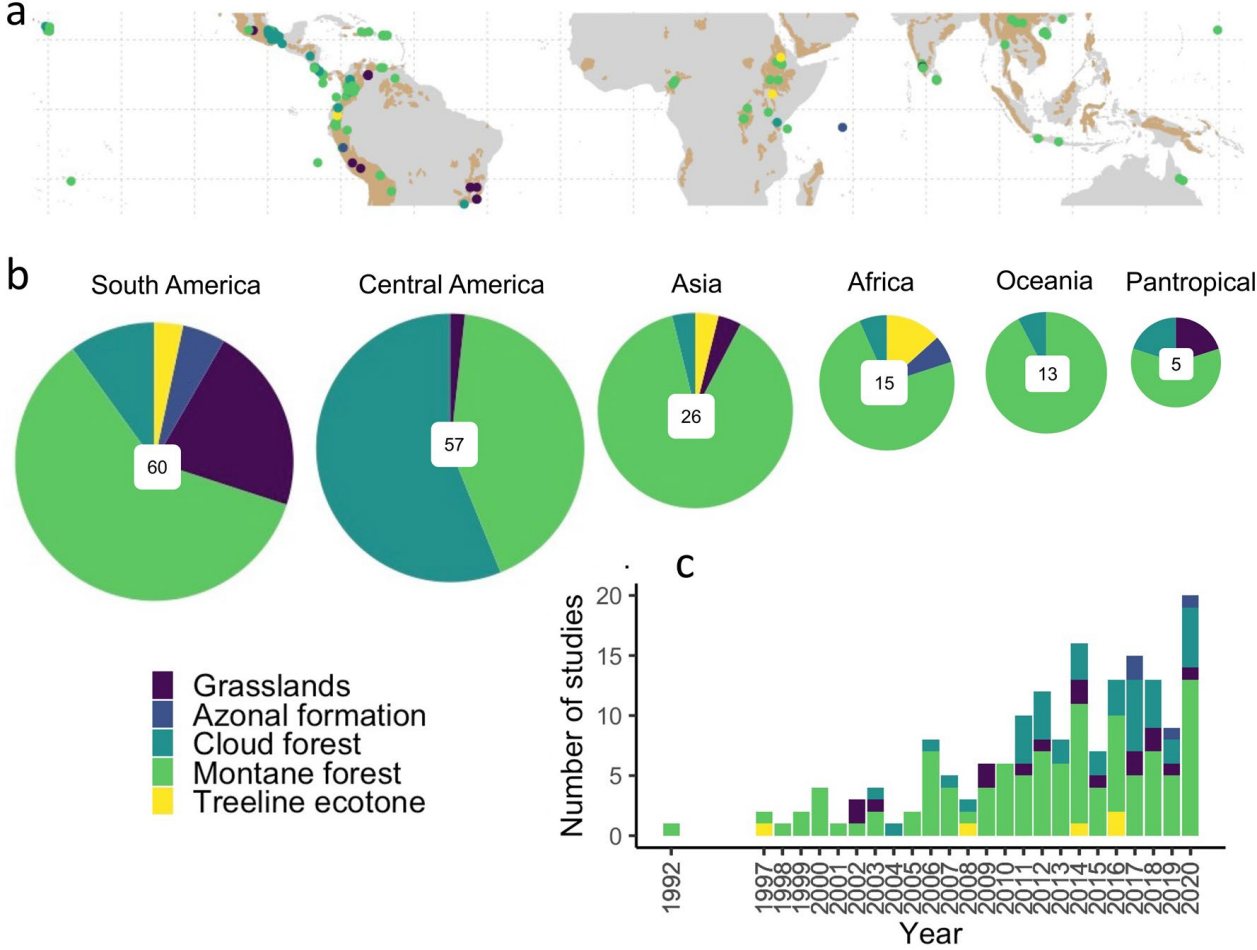
Steady increase in TME studies since the 1990s with a focus on Latin-America and forested mountain ecosystems. (**a**) Location of studies in the different mountain ecosystems in relation to the tropical mountain ranges. Mountain shapefile data from Global Mountain Biodiversity Assessment^43^, base map from R package ‘maptools’^44^ http://CRAN.R-project.org/package=maptools). (**b**) Ecosystem type pie charts for each geographic region scaled in descending order of total number of studies (number in middle of each pie chart). Pantropical refers to studies carried out in multiple geographic regions, c) Number of studies over time for each ecosystem. All figures were generated in R studio version 3.6.2^45^ and using the package ‘ggplot2’^46^.

Most TME restoration studies came from study locations in Central and South America (67%, Fig. 1a). Mexico was the most represented country with 18% of all studies, followed by Colombia (13%) and Costa Rica (9.3%).

Half of the Latin American studies took place outside the large mountain range of the Andes, particularly in small Central American mountain ranges such as the Talamanca Mountains (Costa Rica) and Sierra Madre Oriental (Mexico). This Central American focus mirrors the common trend in tropical ecology, where most science continues to come from a few concentrated research locations.

TME restoration studies in Africa were scarce (only 9% of studies, Fig. 1b), despite the occurrence of prominent mountain ranges such as the Ethiopian and Cameroon Highlands, Tanzanian Eastern Arc mountains, or Mt. Kilimanjaro. Similarly, few studies were conducted in Asian tropical mountains (5% South Asia and 8% in South-East Asia), despite the existence of extraordinarily biodiverse and highly threatened mountain ecosystems such as montane grasslands in the Western Ghats^41,42^.

For Mexican TME studies, 97% of first authors came from Mexican institutions. For Costa Rican TME research, 2/3 of funding and authors came from US based institutions (Supplementary Fig. 1). Funding for Colombian restoration studies was split between Colombian institutions (40%), Global North countries (US, Norway, Germany, UK, together 47%) and supra-national institutions (United Nations, European Union, together 9%).

The number of TME restoration studies remained very low until 2005, followed by a steady increase, reaching maximum numbers in 2020 at the onset of the start of the UN decade of restoration (Fig. 1c). Restoration studies in montane forests were predominant throughout, and almost the only ones before 2000. Since 2004 studies in cloud forests and mountain grasslands increased, while studies about the treeline and azonal formations remained anecdotal through the entire period.

### Strong focus on montane and cloud forests

From an ecosystem perspective, we found a strong focus on forested ecosystems with 62% of studies looking at montane forests, 24% of the studies focusing on cloud forests, 9% on grasslands and even smaller percentages for other azonal ecosystems (Fig. 1b). While this can be explained by the fact that forested tropical mountain ecosystems comprise more than four times the area of mountain grasslands (~ 3,500,000 km^2^ vs 846,286 km^2^, see Table 1), the flipside of this ’forest focus’ is a scarcity of restoration studies in montane and alpine grasslands, many of which rank among the most biodiverse and endemic ecosystems in the world^18,47–49^.

The current focus on reforestation and tree planting in the international restoration agenda^27,50^, exemplified by large international tree planting commitments such as Trillion Trees^51^ and the Bonn Challenge^28^, could also contribute to this dominance of forest restoration studies. Less than a dozen studies were carried out in the vast expanses of the *Páramos* and *Puna*^52–58^ and a few studies in the Brazilian *Campos Rupestres*^48,59,60^ and Western Ghats^41,42,61^. We found only 5 studies (2.8%) on restoration in the tree line ecotone^6,37,62–64^.

We found theme-specific geographic hotspots, such as Veracruz (Mexico) for studies on cloud forest recovery and restoration in abandoned pastures^65–70^. In Hawaii, Mauna Kea was a hotspot for restoration research in montane forest around management of invasive feral pigs^9,71–76^. Finally, eastern Africa was a hotspot around agroforestry and productivity restoration on cultivated mountain slopes^6,77–79^.

### Bias towards short temporal and small spatial study scales

**Figure 2:**
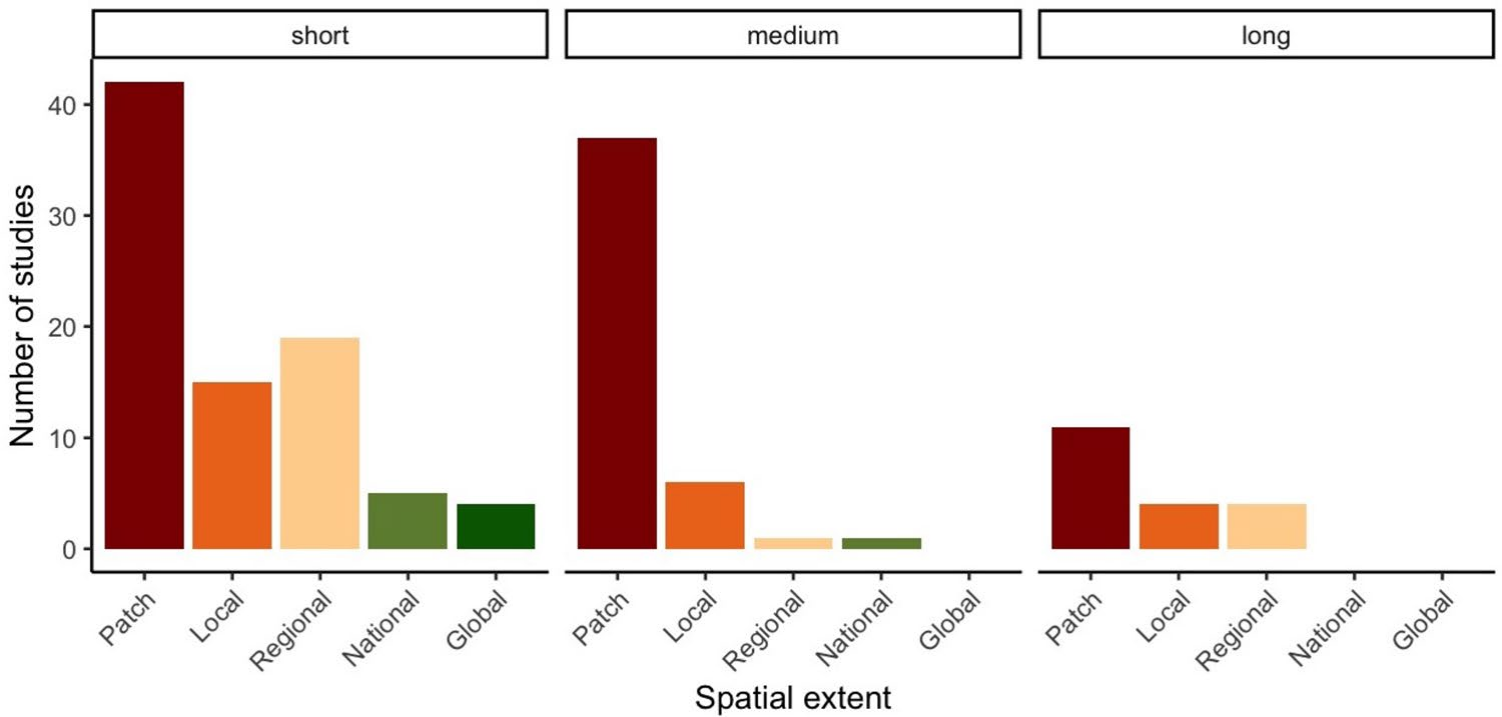
Most TME studies are largely conducted on small spatial scales and in the short term. Spatial categories are: Patch: 10–10^2^ km^2^, Local: 10^2^–10^3^ km^2^, Regional: 10^3^–10^5^ km^2^, National: 10^5^–10^6^ km^2^, Global: > 10^6^ km^2^. Temporal categories are short term < 1 year, medium term = 1–5 years, long-term: > 5 years. Figure generated in R studio version 3.6.2^45^ and using the package ‘ggplot2’^46^.

We characterized studies based on the time scale they look at (short < 1 year, medium 1–5 year, long > 5 years) and the spatial scale of the restoration project (patch scale < 10 km^2^, local scale 10-100 km^2^, regional scale 100–10,0000 km^2^). Most TME restoration studies were short in time and small in space (Fig. 2). Most of the studies (57%) were conducted in the short-term and in the mid-term (30%). Only 13% of studies were long-term with only six studies lasting more than 20 years^6,80–84^, all of which were in cloud or montane forest. Over 33% of the studies were on a patch scale followed by local-scale studies and regional scale studies. There were only 5 pantropical/global assessments, which drew comparisons of restoration processes across distant mountain ranges or across continents^38,85–88^.

These findings are in line with trends in tropical forest restoration, where *“neither the scale of scientific studies nor the restoration projects being implemented have matched the ambitious forest landscape restoration plans that are being proposed”*^89^. The small spatial scales, often at the stand level, show that TME restoration science is so far a patchy-small scale endeavour.

While most studies were primary research including fieldwork, secondary research made up less than 15% of all studies, mostly as reviews^86,90–92^, reports^93,94^, model studies^88,95,96^ and as five remote sensing studies^6,61,81,97,98^.

### Dominance of ecological goals and metrics

**Figure 3:**
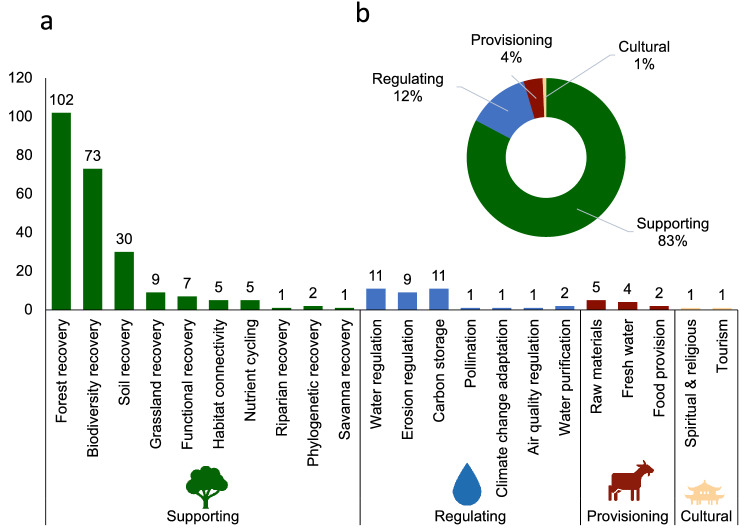
Dominance of supporting ecosystem services as restoration goals. Goals (ecosystem services) and objectives (measurable targets) of restoration studies (**A**) number of objectives for each ecosystem service goal (**B**) grouped into the four ecosystem service goals. Figure generated in Excel.

Most of the reviewed restoration studies had an ecological focus, with over 83% of studies addressing ecological goals and research questions. Most restoration studies aimed to recover supporting ecosystem services, with forest structure recovery being the most frequent goal, followed by recovery of a species or a combination of species (i.e., biodiversity recovery) and soil recovery (Fig. 3). Regulating ecosystem services, especially water and erosion regulation were targeted due to the importance of mountain areas in providing and regulating the hydrological cycle^99,100^. Plant community variables such as plant species diversity, vegetation structure and plant recruitment were the most frequently studied (Supplementary Fig. 2a,c)^87,101–104^.

Over 39% of the studies assessed a combination of biotic and abiotic variables or a combination of vegetation and faunal components and as such took a more holistic ‘ecosystem approach’ (Supplementary Fig. 2b)^105–109^, and 14% of studies assessed animals as part of restoration efforts (Supplementary Fig. 2c), such as bird communities^88,110–112^ or arthropods^113–116^.

While many studies assessed compositional variables (such as species richness, diversity and composition of vegetation structure) the study of functional traits and functional diversity was comparatively underrepresented. In highly diverse ecosystems the inclusion of functional ecology in restoration assessments, such as through measurements of functional diversity, has been shown to better predict restoration success and trajectories than vegetation composition^117^. Incorporating functional trait assessments in restoration research might be especially relevant in tropical mountain ecosystems, due to the strong influence of climate change and the need of species to migrate upslope to track temperatures^118,119^. For example, in a Nigerian montane forest dispersal mode and seed traits of the forest source population played a large role for the colonization of adjacent naturally regenerating pastures, with small animal-dispersed red seeds being dispersed more often and the furthest^120^. Hence, to passively restore degraded forest, the functional-trait composition of adjacent parent populations should be studied to determine colonization potential.

Only two studies aimed at restoring cultural ecosystem services and only few studies involved communities or looked at socio-economic variables. This neglect of socio-ecological dimensions is in line the current underrepresentation of social outcomes and economic cost calculations in restoration of other tropical ecosystems^121^. The international principles and standards for ecological restoration by the Society for Ecological Restoration^122^ highlight that restoration needs to ‘*effectively engage a range of stakeholders, and fully utilize available scientific, traditional, and local knowledg*e’, as the integration of diverse types of knowledge helps improve ecological, social, and cultural restoration goals. Local and traditional ecological knowledge can aid with species selection, identification of successional trajectories and species interactions, as well as the right choice of management strategies involving cultural practices from prescribed burns to grazing management^122^. Some reviewed studies made use of local ecological knowledge by consulting local communities about values and preferences for tree species^123^ or about land use legacy and age of study sites^6,112,124,125^. Only a few studies included economic calculations to estimate cost and/or revenue from timber of restoration plantings^71,88,126,127^.

### Initial degradation due to agriculture and pasture use

**Figure 4:**
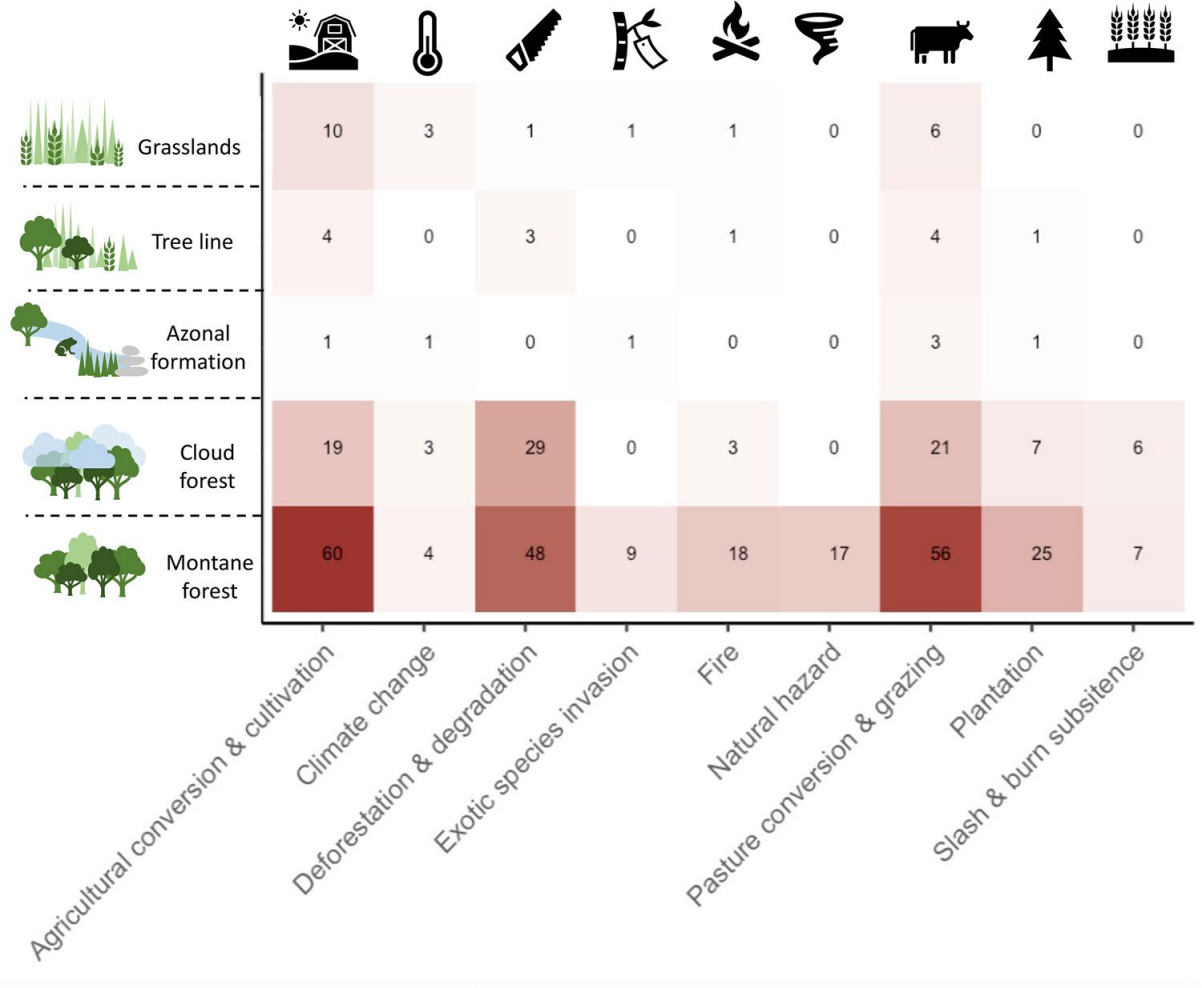
Agriculture, pasture conversion and deforestation are main drivers of initial degradation across TME. Displayed are selected driver of degradation (mentioned more than 10 times across ecosystems) in each TME (for all drivers of degradation see Supplementary Fig. 4a, analogous plot for effects of degradation can be found in Supplementary Figs. 3 and 4b). Figure generated in R studio version 3.6.2^45^ and using the package ‘ggplot2’^46^.

Across all TME initial ecosystems degradation occurred mostly due to agricultural conversion and cultivation (53% of all studies), pasture use (51%) or deforestation and degradation (e.g., logging, clearing, selective logging etc., 46%) (Fig. 4). Plantation use, fire and natural hazards played a substantial role in degradation, too (10–19% of studies). In most studies initial degradation resulted from a combination of multiple degradation causes.

Initial degradation is however site-specific and result in intricate ecological effects posing barriers to restoration, ranging from faunal and vegetation changes, to modified soil and hydrology (Supplementary Figs. 3 and 4b). Arrested succession, i.e., an ecosystem being halted in an early successional state, was a prominent effect of degradation addressed across TMEs^56,62,105^. Reductions in species, functional or genetic diversity or vegetation changes or reductions in vegetation cover or structure were a direct result of initial degradation and particularly prevalent in the highly threatened cloud forests and in montane forests^105,107,128–130^.

### Natural regeneration and seedling planting dominate restoration interventions across TME

**Figure 5:**
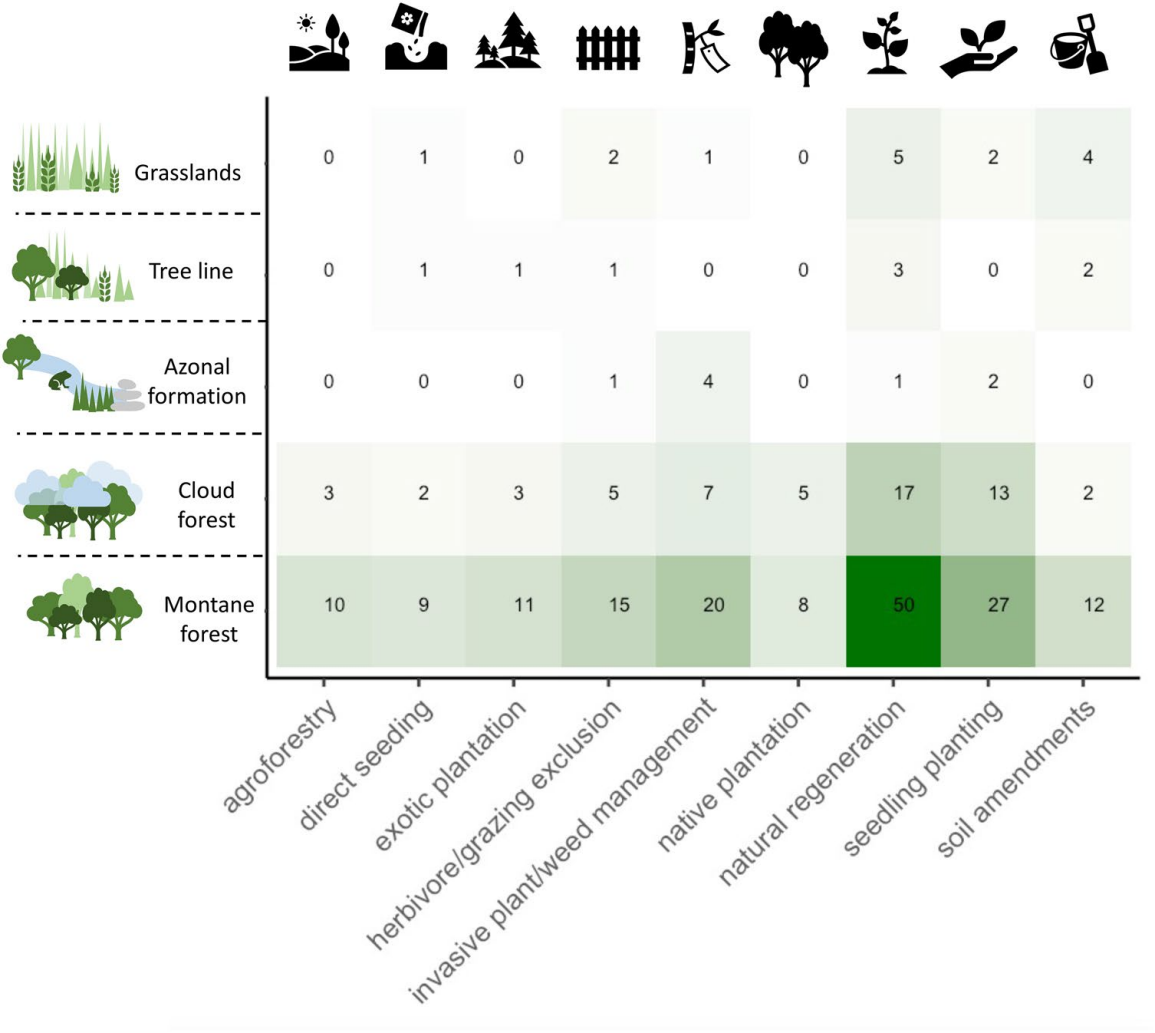
Across TMEs natural regeneration is the most studied restoration intervention, followed by seedling planting and invasive management. Displayed are selected restoration interventions in the tropical mountain ecosystems (mentioned more than 10 times across all ecosystems, see Supplementary Fig. 5a for all restoration methods). Figure generated in R studio version 3.6.245 and using the package ‘ggplot2’^46^.

Across all TME, natural regeneration was the intervention most frequently studied (43% of all studies), followed by seedling planting (25%) and invasive plant management (18%) (Fig. 5 and Supplementary Fig. 5) and often a combination of multiple restoration interventions was investigated.

Seedling planting was mainly deployed in cloud forest and montane forest (Fig. 5), mostly using a few plant families (Supplementary Fig. 5b): *Fabaceae* to enrich the soil with nitrogen, *Myrtaceae* (especially *Eucalypts*) because of fast growth traits and suitability for plantation growth^8^ and *Fagacea*e because of their high conservation value in Costa Rica and Mexico^131–134^. 75% of active restoration studies used exclusively native plant material, 17% used non-native material and 8% studied a mix of native and non-native plants (Supplementary Fig. 6a). Non-native plants were introduced due to easy acquisition, economic viability, fast growth, and facilitative effects for native forest recovery^135^. In 44% of active restoration studies restoration vegetation was animal dispersed, and in 34% wind dispersed (Supplementary Fig. 6b).

Plantations were used as a restoration intervention in cloud and montane forests, with exotic plantations frequently studied in montane forest (Fig. 5). Direct seeding of species and enrichment planting was most frequently studied in montane forest. A host of additional experimental methods were tested only a few times in the TME restoration studies, such as topsoil, seed bank and hay transfers in the mountain grasslands of the *Campos Rupestres*^48,108^, applied nucleation, assisted migration^136,137^ and inoculation of cloud forest seedlings with arbuscular mycorrhizal ^38,138^ (Supplementary Fig. 6a).

### Active and passive restoration in mountain forests following agricultural degradation

In cloud forests, deforestation, pasture use and live-stock grazing caused harsh abiotic conditions following land abandonment and competition by exotic pasture grasses hindered vegetation recovery^76,124,139,140^. Natural regeneration was the restoration method most often studied in these ecosystems, followed by seedling planting and invasive management.

In montane forests, livestock farming and agriculture create forest-pasture mosaics where forest recovery is limited by seed dispersal, competition with exotic pasture grasses, seed predation and herbivory, and unfavourable site conditions^141^. This required active restoration interventions through tree or shrub seedling planting^141^, nucleation planting^11^ or establishment of perching structures for seed dispersing birds^142–144^ to jointly overcome biotic and abiotic limitations^141^. When natural hazards like hurricanes or landslides^145^ caused soil erosion, restoration in montane forests was focussed on recovering regulating ecosystem functions, such as erosion regulation, water provision and hazard prevention through catchment management^135^, restoration of vegetation cover through revegetation and afforestation^101,135^ and natural regeneration^146^. In montane forests of Hawaiian Acacia invasive species, especially feral ungulates caused degradation. Hence, restoration interventions aimed at recovering native biodiversity through a mixture of invasive control, fencing and landscape zonation^72,75,147^.

In TMEs at lower, more accessible and inhabited elevations, restoration often involved land-sharing approaches such as integrative agroforestry practices, through creation of live hedges and fences on working lands^78,148^, underplanting of seedlings in cardamom plantations^149^ or on-farm tree planting to improve ecosystem service provision^150^. On the other hand, in ecosystems less favourable for human inhabitation, land sparing approaches to restoration seemed more common, for instance natural regeneration of cloud forests in pastures^115,151–153^.

### Intense active restoration in grassland following strong land use legacies

Agricultural conversion most prominently caused initial degradation in mountain grasslands (Fig. 4), followed by pasture conversion for alpacas and/or cattle, quarrying and mining, and climate change (see Supplementary Fig. 4a with all degradation drivers). This resulted in strong biodiversity and vegetation change, soil and hydrological constraints (Supplementary Fig. 4b).

Commonly, an intense land use legacy reduced restoration success in mountain grasslands—there seemed to be a disturbance threshold beyond which the damage caused by the disturbance was irreversible, mostly related to the soil being too disturbed for native vegetation to recover^52,154^ or due to low seed dispersal^48,108^.

Natural recovery was generally poor in mountain grasslands, and more intrusive active restoration interventions were needed to restore ecological functions following degradation, such as soil amendments or species introductions. In the *Páramos* centuries of fallow agriculture coupled with overgrazing left the soils depleted and low in nutrients. Soil organic matter and fertility were restored through necro mass incorporation, manure fertilization and transplantations of mats of nurse plants to provide seed sources and improve soil quality^56^. Likewise, in the Brazilian *Campos Rupestres*, quarrying and mining led to soil and vegetation losses and species invasion, hindering natural regeneration and demanding more intense restoration methods^48,108^. However, even hay or topsoil transfer still proved unsuccessful in restoring the native grassland communities^48,108^. Similarly, intense restoration methods were trialled and recommended to restore ancient grasslands in the Western Ghats, which suffered species extinction and habitat loss following tree invasion from exotic forestry plantations^41,61,155^. For this purpose Arasumani et al.^61^ used remote sensing to assign priority areas for restoration and invasive tree removal. Their study was the only mountain-grassland restoration study to date that used remote sensing to inform grassland restoration and showcased a promising avenue of using imagery classification for restoration management.

Despite the apparent difficulty to restore mountain grasslands, the knowledge of tropical mountain grassland restoration seems to be at an early stage compared to tropical mountain forest restoration. Further research will be needed to overcome the barriers in mountain grassland restoration, find cost-effective restoration techniques, and create grassland restoration protocols and knowledge databases.

### Mixed success of the most studied restoration interventions

**Figure 6:**
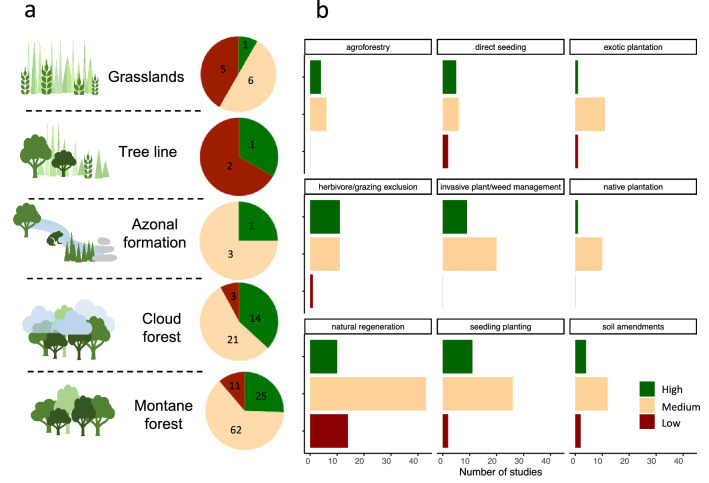
Mixed success rates across restoration interventions. (**a**) Success rates of restoration in each ecosystem. (**b**) Success for each of the most prominent restoration interventions (more than 10 times studied) across all ecosystems. All figures generated in R studio version 3.6.2^45^ and using the package ‘ggplot2’^46^.

We studied restoration success of studies based on how many of the specified objectives (defined in Fig. 3) a restoration intervention achieved (low success = no objectives achieved, medium success = some but not all objectives achieved, high success = almost all/all objectives achieved). Objectives ranged from recovery of biodiversity, soil functions, water and erosion regulation, pollination to food provision and spiritual objectives. The three most prominent restoration interventions across all TME (natural regeneration, seedling planting and plant/weed management) showed mixed levels of success, with most studies classified as ‘medium success’ (Fig. 6). In cloud and montane forest, more than half of the restoration interventions showed medium success, about 20–30% high success, and about 10% low success (Fig. 6a). Studies with low success were particularly frequent in grasslands and the tree line ecotone.

Of all restoration methods, strategies removing disturbance (invasive plant management or herbivory/grazing exclusion) showed the highest success. Removing invasive feral pigs from Hawaiian montane forest for instance, improved soil conditions and nutrient regeneration and led to large increases in understory vegetation^9^. In Sri Lankan montane forests a mixture of invasive grass removal, creation of fire breaks, protection of individual trees and isolation of seedling root systems from competitors proved to be successful^156^, showcasing that often multiple strategies removing disturbance need to be combined.

Strategies involving planting or seeding had mixed results and a trend towards medium success (Fig. 6b). For instance, in Mexico mid-to-late successional cloud forest Oak seedlings were transplanted into abandoned pastures and species showed survival rates between 50 to 70% and due to lower radiative stress resulting from the prevailing cloud cover^131^. Planting has been concluded to be a good option to restore forest and soil quality, however its success depends highly on land-use intensity, initial soil characteristics and species choice^157^.

Agroforestry interventions such as planting of pasture trees or living hedges showed relatively high success levels (~ 40% high success, 60% medium) and often succeeded in reaching combined goals of reducing erosion, enhancing water quality^78^ and improving soil conditions for natural regeneration^143^. Plantations, whether exotic or native, showed mostly medium success. Natural regeneration showed mixed results throughout, and its success was strongly dependent on the local site conditions, on proximity to forest for seed rain and on surrounding and remnant vegetation^34,158,159^.

Extensively used TME, such as selectively logged or mixed-plantation systems recovered biodiversity and vegetation structure well under natural regeneration^160,161^. Abandoned agricultural land recovered more slowly due to habitat constraints, dispersal limitations or competition, and often assisted restoration interventions through weeding, direct seeding or fertilization are needed^57,162,163^. In heavily disturbed systems such as pastures invaded by exotic grasses, environmental filtering was strong and restoration success was low without active interventions such as seedling planting^164^ systematic planting or soil/seed bank transfers^11^.

Our review showed that active restoration planting generally did not help reach restoration goals more successfully than natural regeneration and a site-specific approach based on landscape and micro-site attributes will be needed in TME to choose adequate restoration interventions, as previously shown for lowland tropical forests^34^. Deciding on an optimal site specific approach requires identifying the local abiotic and biotic habitat factors constraints recovery and weighing off costs and benefits of different restoration interventions in the light of finance, time and labour constraints^163^.

### Seed dispersal and habitat constraints limit restoration success

**Figure 7:**
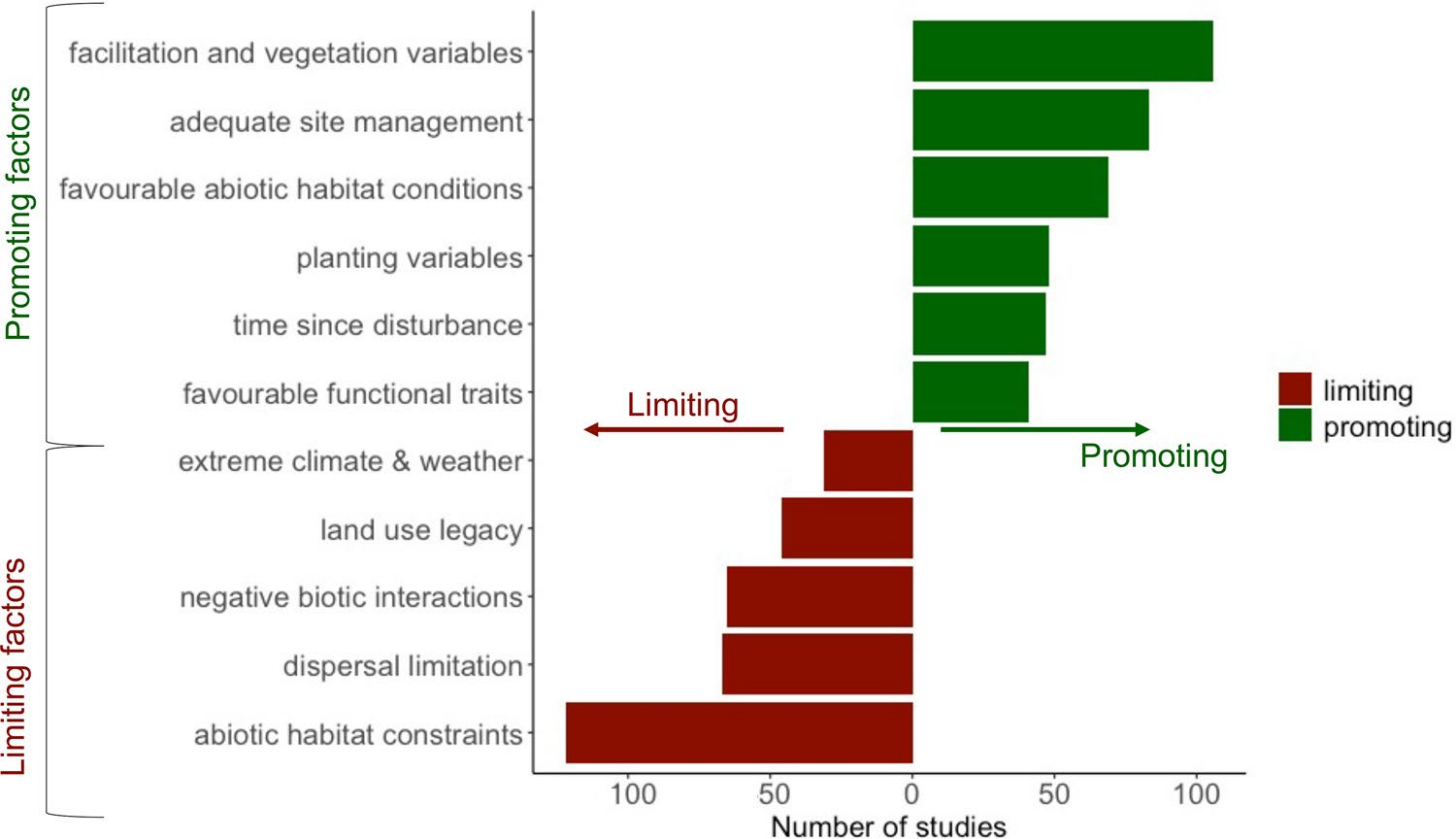
Habitat and dispersal constraints limit restoration success. Factors limiting and promoting restoration success (mentioned more than 30 times across all ecosystems). Factors are arranged on a negative side of the axis if limiting (red), and positive if promoting (green) restoration success (see Supplementary Fig. 7a,b for bar plots of all limiting and promoting factors in each ecosystem). Breakdown of the top three limiting factors: Abiotic habitat constraints encompasses nutrient, water, light and micro-climate limitation, germination, and recruitment limitation. Dispersal limitation encompasses distance from seed source, lack of dispersers, migration limitation. Negative biotic limitations encompass competitive interaction, seed predation, herbivory, pest and disease. Breakdown of the top three promoting factors: facilitation and vegetation variables include structural complexity, proximity to reference habitat, connectivity, remnant vegetation, intra- and interspecific facilitation. Adequate site management includes invasive removal and control, herbivory control, agricultural management, disturbance removal, site protection etc. Favourable abiotic habitat conditions includes beneficial micro-climate, soil conditions, litter properties, light and water availability etc. (see Supplementary Table 3 for all categories). Figure generated in R studio version 3.6.2^45^ and using the package ‘ggplot2’^46^.

Restoration success was mostly limited by abiotic habitat constraints and seed dispersal (Fig. 7). Habitat constraints arised as a result of the harsh mountain environments, with low air temperatures and daily temperature amplitudes that often exceeded seasonal and annual variation^63,79^, recurring frosts^165^, as well as erosion processes due to strong rains, winds, and landslides^78,166^. These factors, in combination with a naturally rugged topography, contributed to acute losses of vegetation, shallow soils and depleted soil seed banks, making it difficult for vegetation to establish^146,167^. These factors often resulted in recruitment limitation^8,87,168–170^.

Limitations due to seed dispersal—the most common form of seed dispersion in TMEs^159,171^—were exacerbated by habitat loss, fragmentation and degradation which disrupt seed dispersers’ abundances and movement pathways^65,83,136,172,173^. Further, negative biotic interactions such as competition between grasses and ferns, pests and diseases, as well as herbivory and seed predation compromised restoration success.

The top promoting factors for restoration success were facilitation and vegetation composition and structure that promotes plant establishment and growth, for instance structural complexity of vegetation, remnant vegetation and proximity to natural habitat. As part of this many studies specifically mentioned facilitation processes which ameliorate micro-environmental site conditions and often contributed to increased restoration success^54,104,174,175^. Facilitative interactions were deliberately employed in restoration studies, e.g. through applied nucleation tree island planting^11,172^, exotic plantations to recover native understories^129^, bracken ferns as facilitators for late succession tree seedlings^128^ or planting to attract seed dispersers^120,140,141,172^. Moreover, site management variables related to removing disturbance, such as eradication of invasive species^84,176^, and protection of restoration sites^6,177^ were mentioned as promoting success. Furthermore, planting variables associated with the right choice of local planting methods, ranging from appropriate seed bank transfers^11^, shade tree^178^ and multi-species planting^161^, and direct seeding^163^ helped improve restoration outcomes.

### Restoring the distinct nature of TME in the light of climate change

Climate change will irreversibly change the ecology of many of the world’s tropical mountains^179^. Mountain ecosystems are projected to undergo ‘elevation-dependent warming’, a process by which the rate of warming is amplified with elevation^180^, resulting in new climate niches. At the same time, many established species will track temperatures through upslope migration, as observed in the Andes^181^. Climate-related limitations such as habitat and recruitment constraints are already a prevalent limiting factor across many of the reviewed restoration studies and may be exacerbated with progressing climate change and its interaction with land use changes^182^.

This will require designing tailored restoration interventions based on the expected eco-climatic changes for a given TME. However, only four montane forest studies and three cloud forest studies reviewed specifically addressed climate change as an initial degradation factor (Fig. 3). Tropical montane cloud forests, for instance, are expected to experience shifted cloud and precipitation distributions, resulting in tree mortality and altitudinal migration^183^. This may require strategies such as assisting species to migrate upslope to track temperatures^137,184^. Many endemic cloud forest tree species have small population sizes, high habitat specificity and low dispersal, due to lack of habitat connectivity, leading to shifted plant-animal interactions due to climate change which will need to be considered under future climate change scenarios^137^. In mountain grasslands, drought is forecasted to intensify, and a functional eco-physiological approach will be needed to design conservation actions^118^.

There are still large research gaps in the context of restoration under climate change, such as assisted migration and germination potentials for a most species and studies on an ecosystem-by-ecosystem basis on climate change implications for TME restoration. Further, the creation of databases with functional traits that are key to climatic tolerance for tropical mountain plant species will help design restoration interventions that leverage facilitative effects and biotic interactions to improve micro-site conditions and provide local refugia.

## Future directions for research

While mountains have long been targeted for conservation due to their unsuitability for agriculture and other forms of anthropogenic use (‘High and Far’ bias of protected areas^185^), the UN Decade of Restoration provides a crucial policy window for large-scale restoration beyond tree planting and for the incorporation of many diverse kinds of ecosystems and restoration approaches.

This synthesis is the first of its kind to show the prevailing trends in geographical and ecosystem bias in tropical mountain restoration research, highlighting pre-dominant research methods and scales, and reviewing restoration interventions and limiting and enabling factors for restoration success. However, we found large gaps in tropical mountain restoration research, ranging from underrepresentation of non-forested ecosystems and socio-economic restoration goals to lack of use of technologies and scarcity of research on implementation and financial viability of restoration projects. Hence, we devise five directions for future research.

1. Given the overwhelming amount of TME studies on a patch and local scale identified here, research on large-scale restoration^186^ will be needed to scale up mountain restoration to a landscape level. Geo-spatial technologies, such as remote sensing could aid with this, for instance through restoration potential and opportunity assessments for entire mountain ecosystems. Science-based open data initiative like the newly launched portal RESTOR, pave the path for the application of remote sensing for large scale restoration purposes.
2. Studies on the social dimensions of restoration in tropical mountains are still scarce. Making restoration a socio-ecological endeavour is especially critical in dynamic social contexts^187^, such as tropical mountain ecosystem which currently undergoing rapid land use changes and often shared between human use and restoration (e.g. mountain plantations, pastures, and fallow cultivation). Hence, solving questions on land tenure, land abandonment, local preferences, and valued ecosystem services through local participation and partnerships will be key to restoring tropical mountain biodiversity in the long term.
3. Monitoring efforts are a key aspect of the UN decade on restoration^188^. Advancing and applying technologies and monitoring protocols for tropical mountain restoration could help with increasing restoration success for instance through adaptive management. With most reviewed studies conducted in the short-term, long-term restoration trajectories in almost all ecosystems are uncertain. Drone technologies for instance, could provide exciting avenues for recurring monitoring of recovery of vegetation variables on a patch level in inaccessible and remote mountain areas, while also offering opportunities for assisted regeneration via aerial seeding^189^.
4. Thus far, tropical mountain restoration studies that explicitly consider the changing climate in mountains, and tailor restoration interventions to a dynamic future reference state are still scarce. The choice of climate resilient species with the right functional traits will be essential for mountain restoration to thrive under a changing climate. For instance creating ‘functional trait libraries’ of reference ecosystems and understanding traits that underpin resilience in a specific ecosystem context will be useful to guide the selection of species and restoration approaches.
5. The paucity of restoration studies assessing mountain grassland restoration calls for a better inclusion of these underrepresented systems in future research. Despite the critical importance of alpine grassland systems for water regulation, soil maintenance and biodiversity^18^ and their large extent and degradation status there are very few successful restoration studies in alpine grasslands. The creation of alpine grassland restoration networks and restoration protocols could be a solid next step.

We hope this synthesis will help direct research priorities which can contribute to effectively restore tropical mountains in order to help mountain biodiversity flourish, and ensure that communities that depend on their ecosystem services will thrive for decades to come.

## Methods

We followed the SALSA (Search-Appraisal-Synthesis-Analysis) method which includes the steps of (1) Searching for literature; (2) Appraisal to decide which studies are included; (3) Synthesis and (4) Analysis of studies, as this method has been shown to be applicable to assess knowledge trends and gaps in environmental science research^40^. We preferred this method to other established methods such as PRISMA, PICO or BACI, as we review across a wide set of ecosystems, contextual settings, metrics and restoration interventions, and as such decided not to adopt a purely comparative approach^40^.

### Literature search

Documents were searched on the databases Web of Science, Scopus and Science Direct (search date 20/1/2021) and on Google Scholar (search date 3/12/2020). We included papers for the period 1988–2020, as 1988 marks the foundation date of the Society for Ecological Restoration and 2020 marks the last year before the start of the UN decade for Restoration. We included articles written in any language.

We tested different search strings for Title-Abstract-Keyword (TAK) search of varying length and complexity on the four databases, with the goal of finding a search term that returned a high but manageable number of search hits and enabled high relevance of papers for answering the questions of the review (Supplementary Table 1). We decided on a simple search string that could be applied across all databases despite the different search algorithms. Our final search string across all databases included a word related to restoration (restor*), to tropics (tropic*) and to mountain environments (*mountain OR montane OR altitude OR alpine OR andes*) (detailed search string specification in Supplementary Table 1), but no word related to specific TME because the nomenclature across TME is not standardized. We decided to include the word ‘Andes’ as it is often used instead of another mountain-related word. We further did a top up search in Google Scholar in order to include articles in other languages and from smaller national literature databases that might have been missed otherwise (such as Scielo). However, since Google Scholar only allows for full text search as opposed to TAK search, we only included the first 100 search results.

### Appraisal: inclusion and exclusion

Our search string yielded a total of 980 articles, of which 532 remained for screening following manual and automated duplicate removal (Supplementary Fig. 8). First, we performed a title and abstract screening to exclude studies that (1) did not have a focus on ecological restoration sensu Society for Ecological Restoration (2002) and (2) were clearly neither in tropical nor mountain locations. We include studies on all kinds of restoration elements and interventions, including passive restoration (e.g. natural regeneration), assisted recovery (e.g. interventions that involve soil amendments, invasive species management etc.) and active restoration (introduction of species, seedling planting, transfer of seed banks etc.). Second, we did a full-text screening of the 245 selected articles to exclude (1) grey literature and (2) studies with locations outside tropical latitudes (i.e. outside 23.5°N and 23.5°S) and where study locations were not referred to as montane/mountain/alpine or not situated above 1000 m asl.

### Synthesis and analysis

We synthesized and coded studies using Microsoft Excel, where we extracted tags for the following categories: publication year, study site, study type, initial degradation causes, restoration metrics, restoration methods and success. Each of these categories had additional specific sub-categories. For instance, restoration metrics had the categories: restoration goals; studied response variables; taxonomic groups etc. (Supplementary Table 2). Each study could have multiple tags for each sub-category (e.g. for the restoration goals category each study got assigned as many goals as were mentioned/studied).

For the top three countries with the highest number of TME studies (Mexico, Costa Rica, and Colombia) we extracted first and last author affiliations and country of funding source to elucidate on funding flows and potential bias in geographical authorship.

As a measure of success of restoration interventions, we qualitatively assigned each study the categories low (no/almost no objectives reached), medium (multiple, but not all objectives reached) and high (all/almost all objectives reached), based on how many of its defined objectives were achieved (e.g., a native plantation intended to improve soil, water, vegetation structure and biodiversity but only improved soil qualities and structure would be assigned a medium value). We realize this metric is quite simplistic, but it allowed for general detection of trends across a multitude of different restoration studies that measured different goals, had different research designs and restoration variables of interest. For the same reason, we decided not to study effect sizes, as the study designs, treatments and measured target variables differed too much to give us adequate sample sizes for specific comparison.

Data analysis and visualization was carried out in Excel and in R studio version 1.2.1335^45^ and using the package ‘ggplot2’^46^.

## Supplementary Information

Supplementary Information.

## Data Availability

The data that support the findings of this study is publicly available on Mendeley Data (10.17632/gvy63jydyg.1, URL: https://data.mendeley.com/datasets/gvy63jydyg/1).
