# Supplementary material for: A synthesis and future research directions for tropical mountain ecosystem restoration
Source: Sci Rep. 2021 Dec 14;11:23948. doi: 10.1038/s41598-021-03205-y (PMC8671388; doi:10.1038/s41598-021-03205-y)
Supplement: Supplementary file 1 — Supplementary Information. [file 41598_2021_3205_MOESM1_ESM.pdf]

A SYNTHESIS AND FUTURE RESEARCH DIRECTIONS FOR TROPICAL MOUNTAIN ECOSYSTEM RESTORATION

Tina Christmann & Imma Oliveras Menor

I. Supplementary results

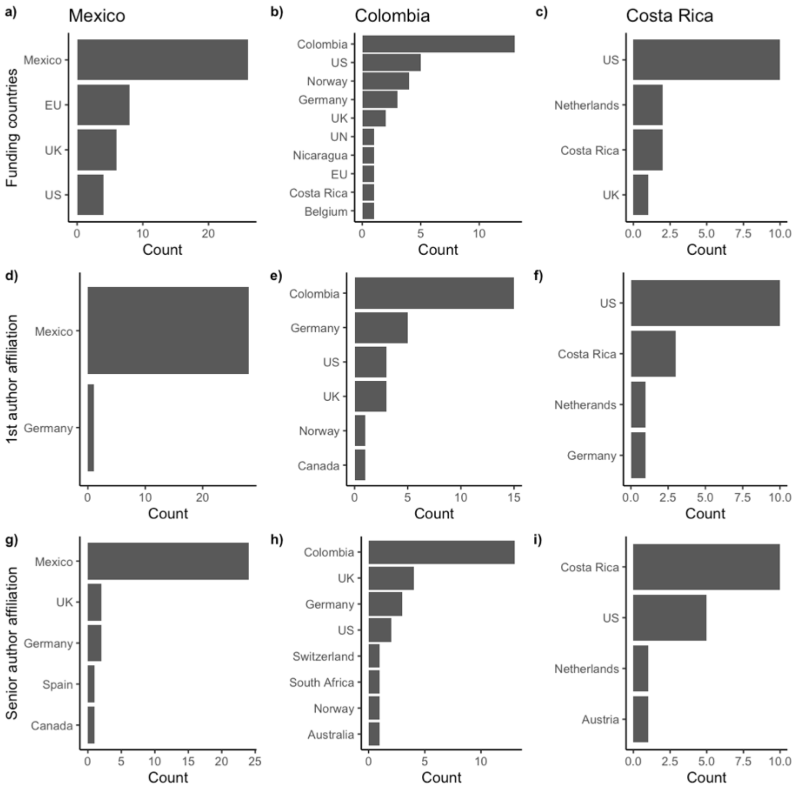

Supplementary Figure 1: Funding & affiliations of studies for the top three countries. a) – c) Funding countries, d) - f) Affiliations first author, g)- i) Affiliations last/senior authors

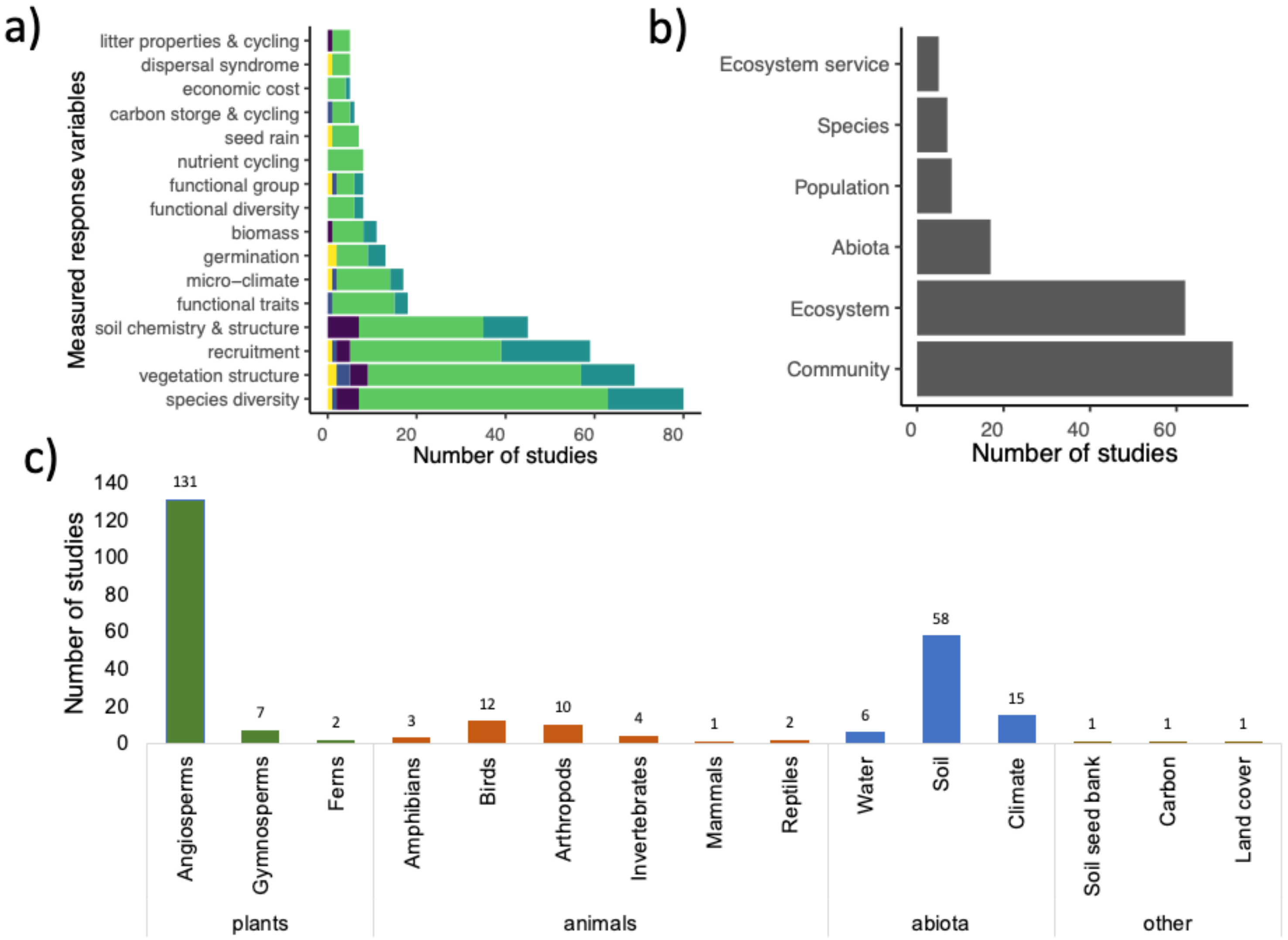

Supplementary Figure 2: A) Measured response variables of restoration studies measured more than 5 times, B) Ecological level of response variable C) Taxonomic group of response variable

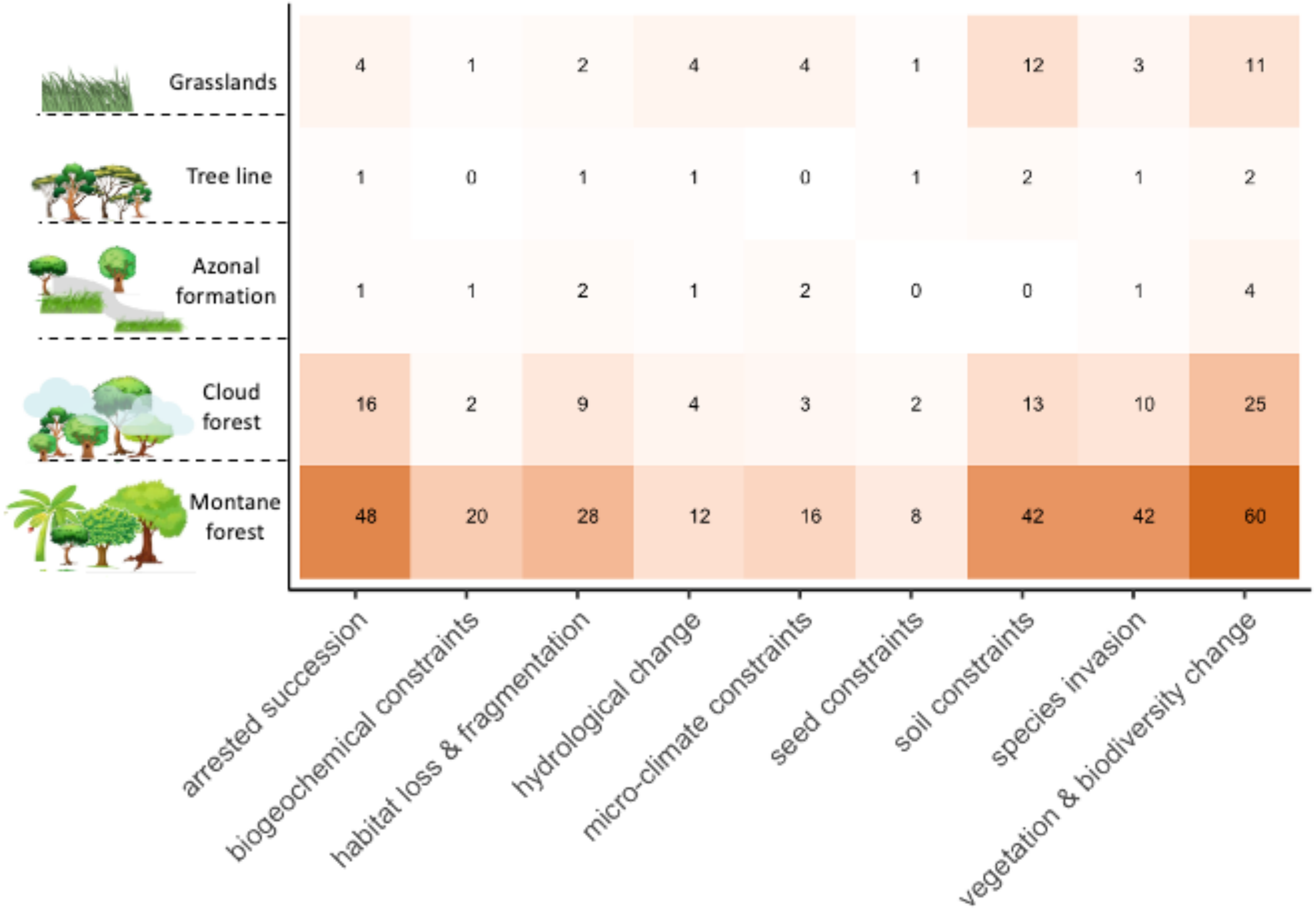

Supplementary Figure 3: Ecological effects of degradation (mentioned more than 10 times) in each ecosystem

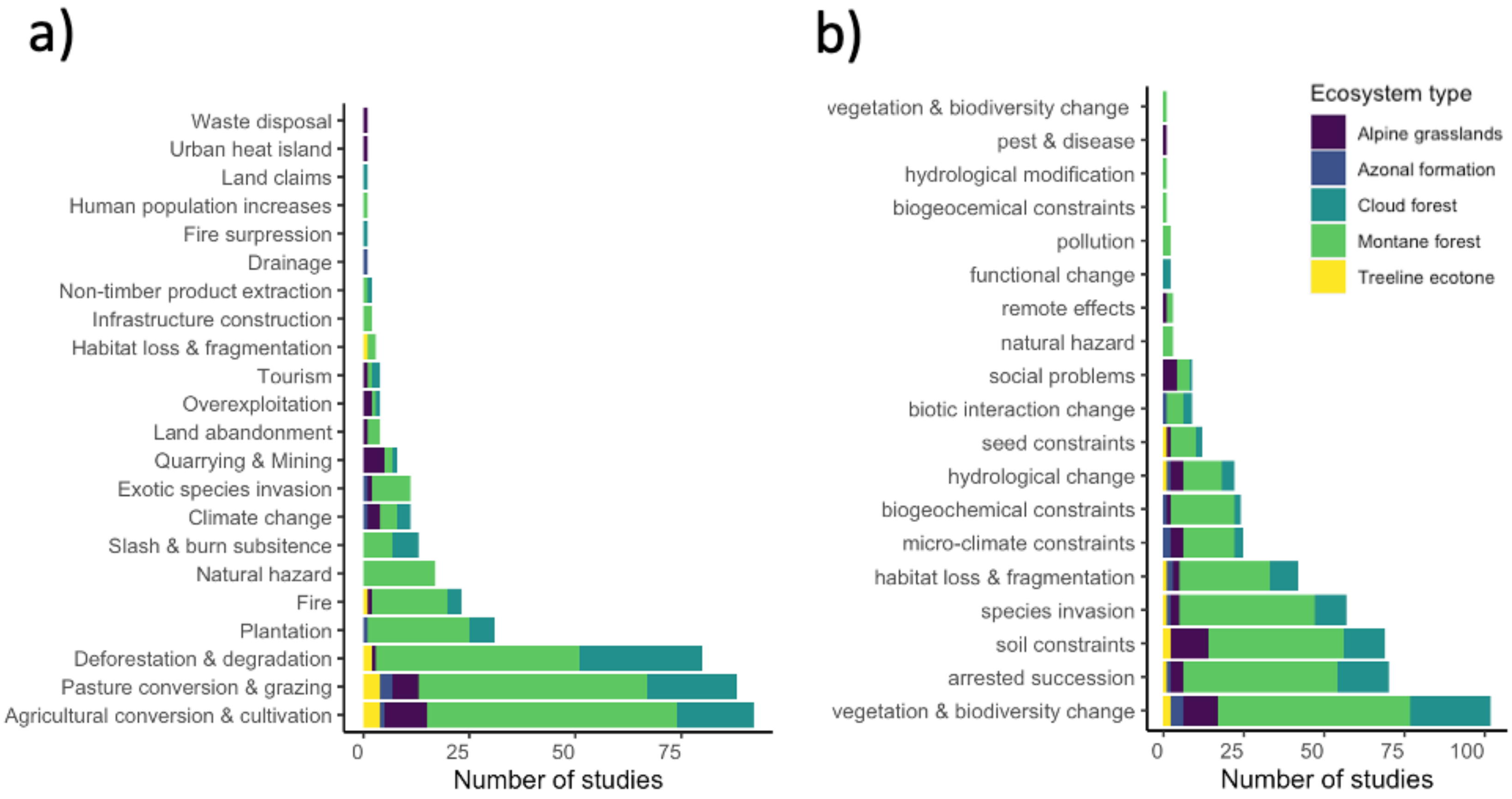

Supplementary Figure 4: a) All drivers of degradation mentioned in studies, b) All effects of degradation mentioned in the studies

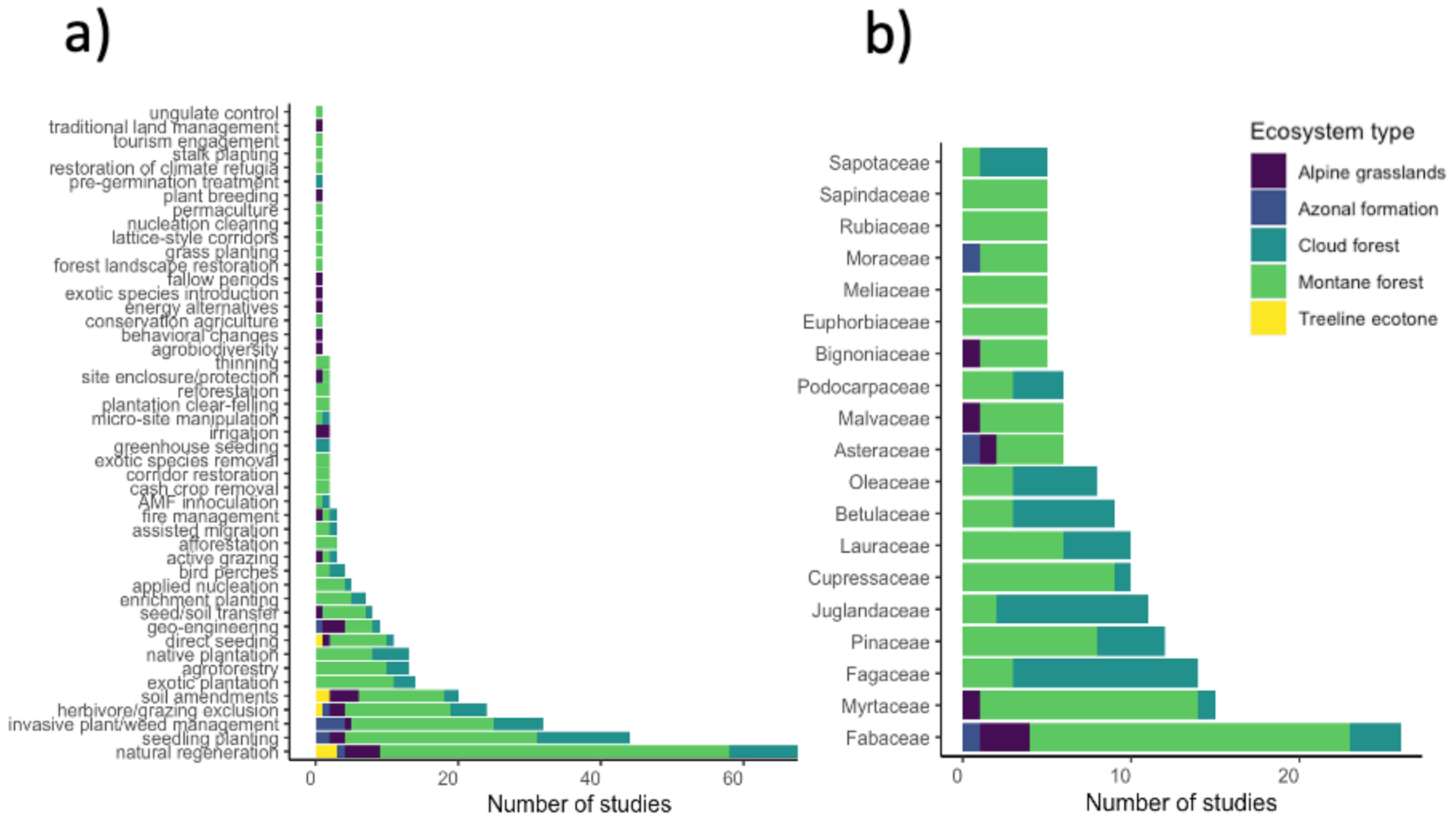

Supplementary Figure 5: a) All restoration methods studied, b) Most frequently planted families (mentioned more than 5 times) in studies with active restoration plantings

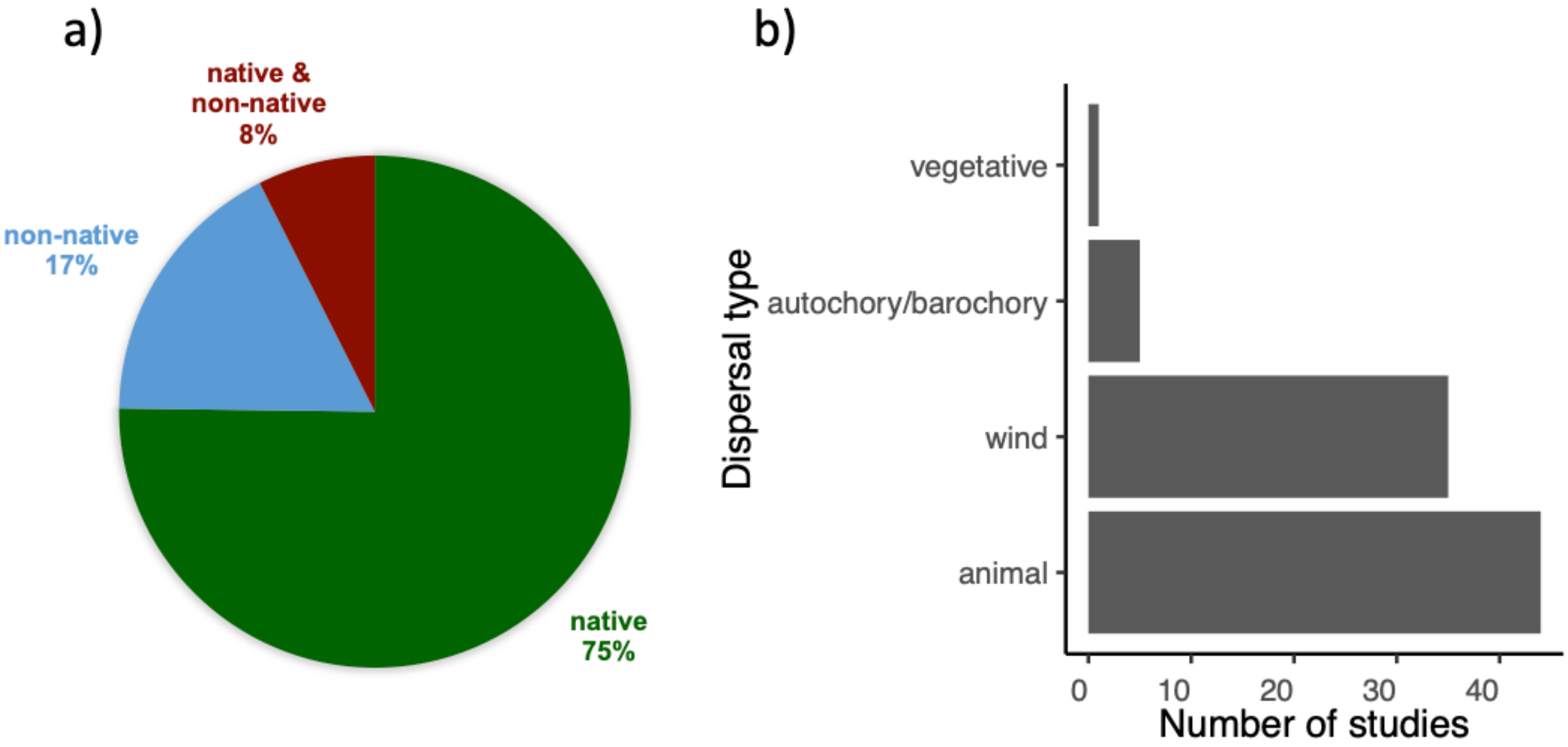

Supplementary Figure 6: A) Origin of planted restoration material and B) dispersal type of planted material

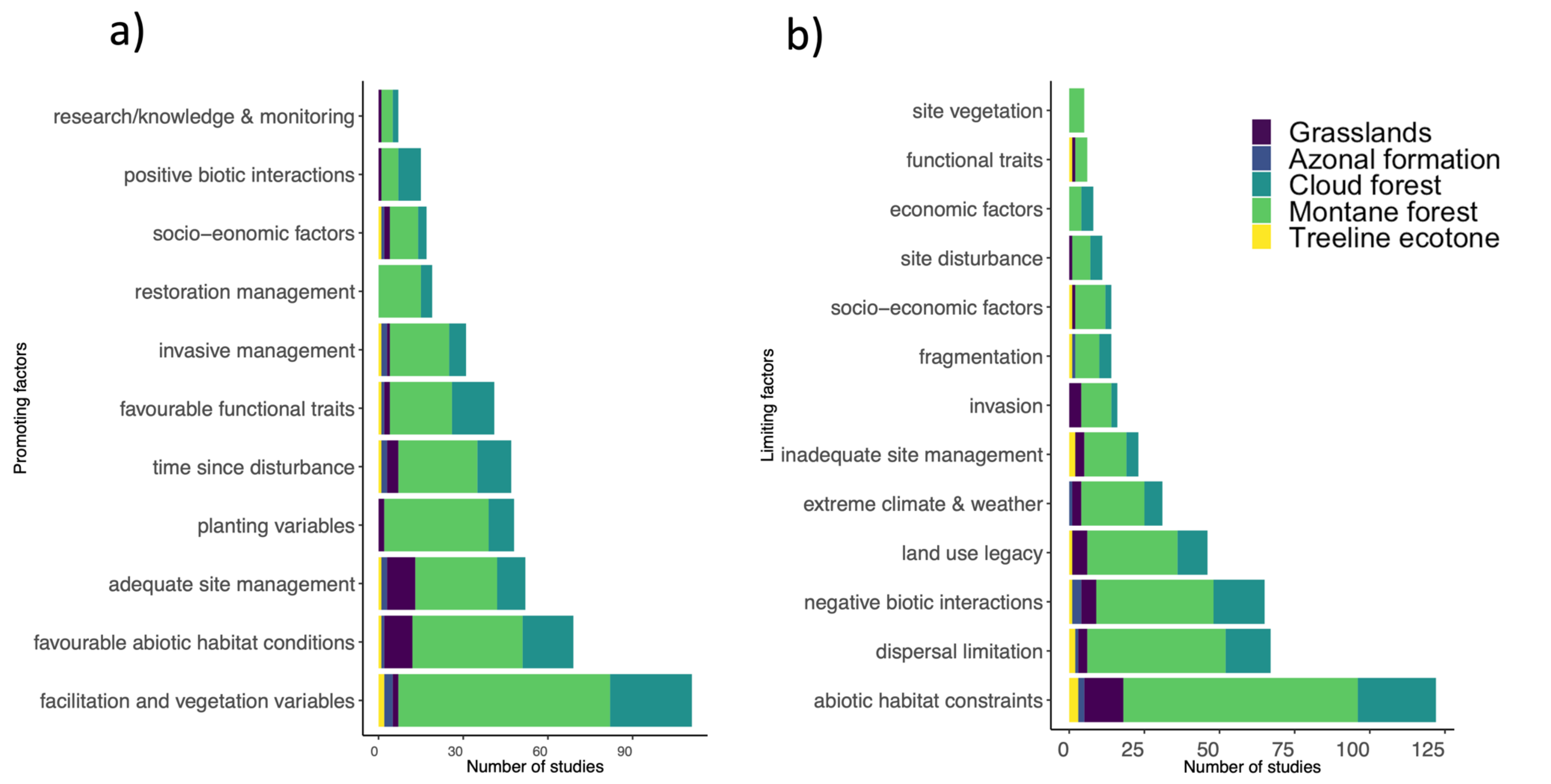

Supplementary Figure 7: All factors limiting or promoting restoration success in each ecosystem. a) Promoting factors, b) limiting factors

II. Supplementary methods

Supplementary Table 1: Tested search strings on the four literature databases, including the number of articles per search string. Final search string is highlighted in bold.

**Database**	**Search terms: in title, abstract and keywords of journal articles (research articles and reviews) from 1988-2020**	**Subject area/Research area**	**No of articles**
Scopus	restoration OR restor* OR rehab* OR recovery AND tropic* AND mountain OR montane OR elevation OR altitud* OR andes OR andean OR andine	Agric and biol., environmental science, earth and planetary science	623
	restoration AND tropic AND mountain OR montane OR elevation OR altitude	Agric and biol., environmental science, earth and planetary science	67
	TITLE-ABS-KEY (restoration OR restor* OR rehab* OR recovery AND tropic* AND mountain OR montane OR elevation OR altitud* OR andes OR anden OR andine AND forest OR grassland OR ecosystem) AND (LIMIT-TO (SRCTYPE , "j")) AND (LIMIT-TO (SUBJAREA , "AGRI") OR LIMIT-TO (SUBJAREA , "ENVI") OR LIMIT-TO (SUBJAREA , "EART"))	Agric and biol., environmental science, earth and planetary science	477
	TITLE-ABS-KEY (restoration AND tropical AND mountain OR restoration AND tropical AND montane OR restoration AND tropical AND altitude OR recovery AND tropical AND mountain OR recovery AND tropical AND montane OR recovery AND tropical AND altitude OR restoration AND andes OR recovery AND andes)	Agric and biol., environmental science, earth and planetary science	10
	**TITLE-ABS-KEY (restor* AND tropic* AND (mountain OR montane OR altitude OR alpine OR andes)) AND (LIMIT-TO (SUBJAREA , "AGRI") OR LIMIT-TO (SUBJAREA , "ENVI") OR LIMIT-TO (SUBJAREA , "EART"))**	**Agric and biol., environmental science, earth and planetary science**	**395**
Science direct	TITLE-ABS-KEY= (restoration AND tropic AND mountain OR montane OR elevation OR altitude)	Agric and biol., environmental science, earth and planetary science	28,981
	TITLE-ABS-KEY= (restoration OR recovery AND tropic AND mountain AND forest OR grassland)	Agric and biol., environmental science, earth and planetary science	31

	TITLE-ABS-KEY= (restoration tropical mountain OR restoration tropical montane OR restoration tropical altitude OR recovery tropical mountain OR recovery tropical montane OR recovery tropical altitude OR restoration andes OR recovery andes)	Agric and biol., environmental science, earth and planetary science	110
	**TITLE-ABS-KEY= (restoration OR restore) AND (tropic OR tropical) AND (mountain OR montane OR altitude OR alpine OR andes)**	**No specification**	**94**
Web of science	TS (topic search, is equal to TITLE-ABS-KEY) = (restoration AND tropical AND mountain OR restoration AND tropical AND montane OR restoration AND tropical AND altitude OR recovery AND tropical AND mountain OR recovery AND tropical AND montane OR recovery AND tropical AND altitude OR restoration AND andes OR recovery AND andes) from 1992-2020	environmental sciences ecology or forestry or geology or plant sciences or biodiversity conservation or agriculture or physical geography or geochemistry geophysics or science technology other topics or meteorology atmospheric sciences or water resources or zoology or biophysics or remote sensing or geography or evolutionary biology	668
	**TS=(restor* AND tropic* AND (mountain OR montane OR altitude OR alpine OR andes))**	**No specification**	**391**
Google Scholar	**restor * tropic * mountain OR montane OR altitude OR alpine OR andes**<br><br>**no TAK search possible, hence high number of unspecific hits and only selection of the first 100 articles**	**No option to specify**	**2160, only first 100 entries selected**

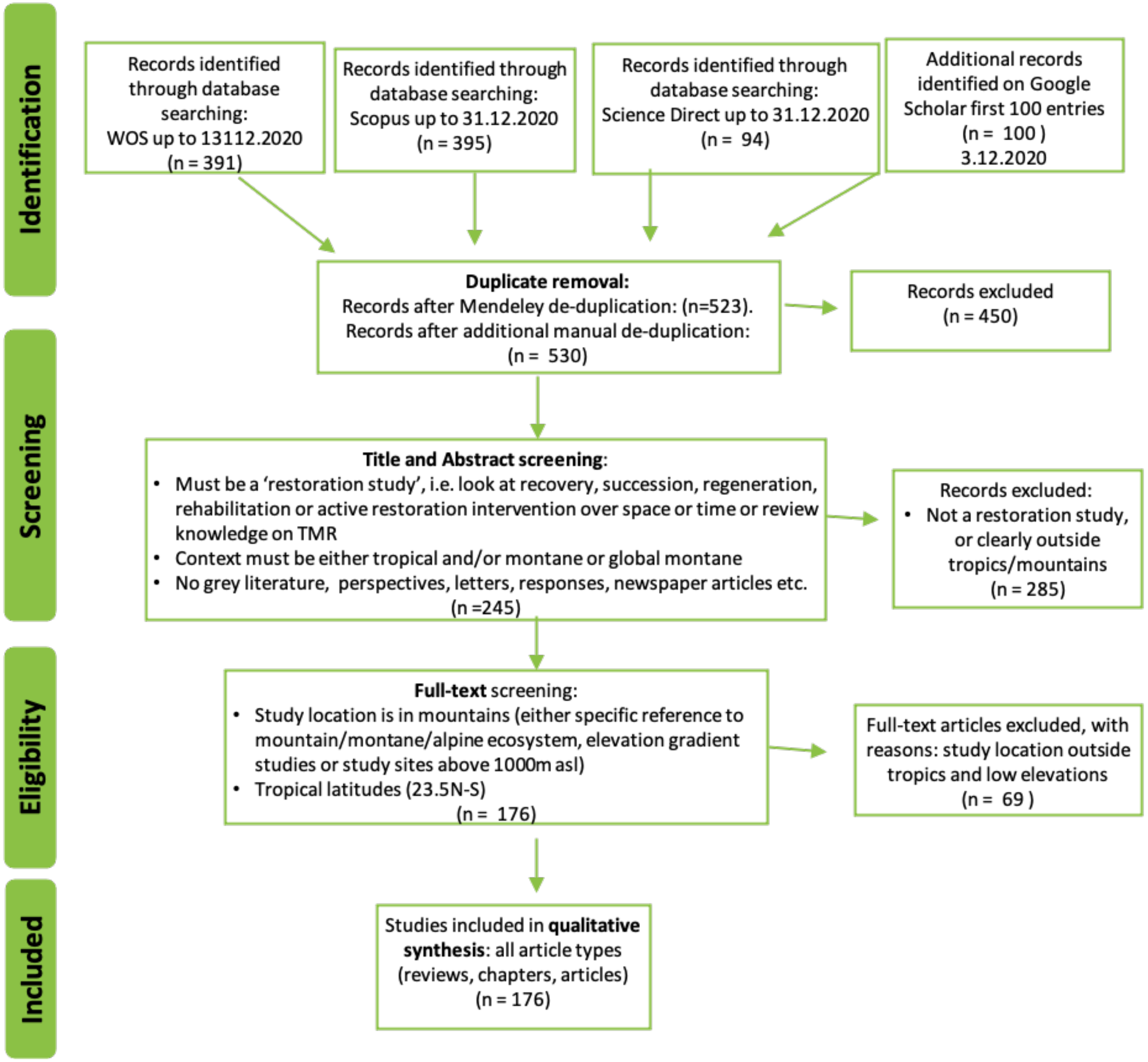

Supplementary Figure 8: Prisma (Preferred Reporting Items for Systematic Reviews and Meta-Analyses) flowchart of study selection

Supplementary Table 2: Table for data extraction from articles

Criteria	Category	Tags
**Paper details**	Study year	Year after 1988
**Study site**	Country	Name of country
	Geographic coordinates	Latitude (-23.5 to 23.5) and Longitude (-180 to 180)
	Geographic region	S-America, C-America, Asia, Africa, Oceania, Australia, Pantropical studies
	Mountain range	Andes, Himalaya, Wester Ghats, Central Cordillera and more
	Ecosystem	5 broad Ecosystem types (Montane forest, cloud forest, treeline ecotone, alpine grasslands, azonal formations), and many subtypes like: upper montane forest, high Andean forest, wet montane forest, dry montane forest, shrubland etc
	Elevation	in m asl
	Climate of site	Mean annual precipitation [mm] and mean annual temperature [° C]
	Protection status of site	IUCN category I-VII
**Study type**	Primary study	Field sampling, in situ methods, experiment, treatment
	Secondary study	remote sensing, modelling, literature review, databases, reports, syntheses…
	Size of study	Patch: $10–10^2$ km^2, Local: $10^2–10^3$ km^2, Regional: $10^3–10^5$ km^2, National: $10^5–^{106}$ km^2, Global: $>10^6$ km^2
	Study duration	Short term (<12month), mid-term (>=1y-5y), long-term (>=5y)
**Degradation type**	Initial degradation cause	Pasture conversion & grazing, Agricultural conversion & cultivation, Deforestation & degradation, Commercial plantation, Human induced fire, logging, selective logging, slash & burn, climate change and more (see Supplementary 7 a)
	Degradation effect	arrested succession, biodiversity change & loss, soil degradation, habitat fragmentation, harsh microclimate, invasion by grass change and more (see Supplementary 7 b)
**Restoration metrics**	Restoration goal	Grouped by ecosystem services (provisioning, regulating, supporting, cultural)
	Response variable measured	Vegetation structure, species diversity, functional diversity, recruitment, hydrology and more (see Supplementary 5 a)
	Successional stage studied ecosystem	Early (0- <=5y), mid (5- <20y), late (>=20y)

	Taxonomic group of response variable	Plant (angiosperms, gymnosperms, other), animal amphibian, birds, arthropods, invertebrates, mammals, reptiles), abiota (soil, water, climate), other (soil seed bank, carbon, land cover)
	Ecological level of response variable	Population, species, community, ecosystem, ecosystem service, abiota (like soil, water and climate)
	Limiting factors	Distance to forest, dispersal limitation, recruitment limitation, unfavourable soil, unfavourable micro-climate and more (see Supplementary 4)
	Promoting factors	Time since disturbance, facilitative effects, favourable micro-climate, remnant vegetation, proximity to forest, functional diversity and more (see Supplementary 4)
**Restoration methods**	Restoration intervention	herbivore/grazing exclusion, invasive plant/weed management, natural regeneration, seedling planting, soil amendments and more (see Supplementary 8 a for all interventions)
	Type of restoration	Active (planting), passive (natural regeneration and site protection), assisted recovery (some controlling intervention)
	Restoration material: dispersal type	Animal-dispersed, wind dispersed, barochory/autochory, vegetative
	Restoration material: origin	Native, non-native, native & non-native
	Restoration material: taxonomy	Plant family (see Supplementary 8 b)
**Restoration success**	Success of restoration intervention	Low (did not reach any of its goals), medium (some goals reached, but not all) and high (almost all or all goals reached)
**Conclusions (qualitative)**	Implications	One paragraph per study with main take away
	Suggestions & recommendations	One paragraph per study with directed recommendations such as restoration methods proposed or critical knowledge gaps that need addressing

Supplementary Table 3: Detailed specification of categories for limiting and promoting factors

	Category	Tags
**Limiting factors**	Habitat constraints	Unfavourable micro-climate/soil conditions, light limitation, water limitation, soil acidity, micro-habitat availability, low seed bank capacity, temperature limitation, absence of soil seed bank, leaching, sandification, recalcitrance, photo-inhibition, unfavourable litter conditions, nutrient excess, nutrient lack, germination limitation, reproduction limitation, species specificity of recruitment, topographic constraints,dessication
	Dispersal limitation	Distance from forest, lack of dispersers, migration limitation, species colonization capacity
	Negative biotic interactions	Competition by fern/grass/trees/other growth forms, intra-specific competition, seed predation, mammal herbivory, insect herbivory, Pollinator limitation, mineralization of SOM,allelopathy, reduced mycorrhiza, fungal attack, pest & disease
	Land use legacy	Previous clearing of forest or unfavourable use of ecosystem, agricultural or pastoral legacy
	Inadequate site management	Inadequate fire/grazing management, poor waste/resource management, fallow management, vegetation removal, herbicide toxicity, afforestation, agriculture in steep areas, annual cropping, unfavourable exotic species plantation,unfavourable bird perches
	Extreme climate & weather	Recurring fires, intense rainfall, rainfall seasonality, climate change, cloud cover changes
	Socio-economic factors	Divergent stakeholder views, lack of local engagement, trade-offs between restoration goals, poor stakeholder management, poor planning documents, human accessibility of site,absence of legal framework, need for personnel,corruption, poor law enforcement, population pressure, Economic cost,limited funding
	Invasion	Invasion by exotic/non-native plants, re-invasion, proximity to invasive sources,
	Functional traits	Unfavourable seed traits, seed production limitation, fast-growth associated tree mortality, seed dormancy, low resource effciency of flora
	Site disturbance	Selective logging, wood harvesting, disturbance intensity level, agricultural disturbance, clearing method, timber extraction, livestock disturbance,planting disturbance
	Site vegetation	Structural homogeneity, unfavourable vegetation structure,canopy gaps, unfavourable belowground plant community characteristics
	Inadequate seed treatment	Physical seed damage, inadequate seed treatment
	Fragmentation	lowland vegetation cover, absence of undisturbed forest, surrounding landcover, matrix type,edge effects, forest loss
	Research constraints	Lack of long-term data, lack of regeneration ecology knowledge
**Promoting factors**	Facilitation and vegetation variables	Facilitation by trees and other growth forms ,Age of tree, complex vegetation structure, remnant trees, remnant vegetation, vegetation cover, dead wood, root biomass, standing dead trees, structural complexity, tree size, suitable canopy cover, insitu propagule sources, floristic diversity, habitat connectivity,proximity to forest, distance from human settlements, distance from human settlement, distance to settlement, proximity to source population, configurational heterogeneity,species turnover, site remoteness, connectivity enhancement, evapotranspiration by vegetation,high phylogenetic diversity on site, water trapping through epiphytes, cloud water nutrient input
	Adequate site management	prescribed fires, management of nurse species, management of exotic plantation, fire management, fertilization,intermediate disturbance, enclosure strength,site protection, disturbance reduction/removal, low deforestation intensity,banning of harvesting, herbivory control,legal site protection, feral pig exclusion, grazing exclusion, land sparing, Fern removal, grass removal/reduction at edge, invasive animal removal, invasive plant removal, pig population control, reproductive control of inhibitor species, removal of invasive seedlings, bamboo control, weed control,competition control

	Favourable abiotic habitat conditions	Adequate micro-climate, light availability, litter amount, soil conditions, thick litter layer, vital soil seed banks, hydrological conditions, elevation and topographic favouring factors
	Time since disturbance	Time since abandonment or since start of restoration intervention
	Positive biotic interactions	AMF inoculation, symbiotic inoculation, biotic interactions, positive dispersal feedback,diverse microbiome,gut passage of seeds
	Favourable functional traits	Bird size, frugivorous birds, trait plasticity, wind dispersal, zoochorous fruits, colonization ability, generalist bird species, N2 fixer, nitrogen fixing trees, sapling drought tolerance, vegetative reproduction, vegetative growth, planting of generalist tree species, functional redundancy, N-fixers,plasticity
	Socio-economic factors	Education, training, targeted outreach, benefits to local communities, community care, development of positive relationship, ecosystem services scarcity path, effective communication, local public engagement, locally perceived need to restore, conservation contract, formal relations building, leadership building, visitor education, crisis restoration,capacity building,communal land, learning by example, legal site protection, personal incentives, Financial incentives, broaden market ranges, carbon financing mechanisms, certification, combination of tourism and restoration, ecotourism, high value species, market-based instruments, socio-economic gain, alternative fuel sources, low agricultural site value, private incentives for reforestation
	Planting variables	planting container size, sapling diversity, shurb planting, shade tree planting, seedling planting, seed tree planting, seed bank transfer, seed addition, reforestation, nucleation techniques, native species planting, mutispecies planting, forest cover restoration, direct seeding, strip planting, ex-situ propagation, vegetation planting, tree planting, seed transfer, initial age of seedlings, large seedling size,enrichment planting,seed burying,root grafting, native species plantations, seedling protection,scarification, seed provenance, generic variation of seeds, frost protection, irrigation, micro-catchment creation
	Restoration management	species-specific approach,site selection, spatial prioritization, use of multi-function value trees, multiple restoration goals, change-adapted restoration planning, biodiversity-carbon-synergies, project specific planning, long rotation times, nitrogen-fixing pasture trees, agrobiodiversity, fallow length, climate resilient crops,agroforestry, water provision technologies,assisted migration,wind breaks
	Research/knowledge & monitoring	Knowledge integration, long-term monitoring, disruptive innovation
	Macro-site conditions	Elevation, rainfall, cloudwater nutrient input,climate change effects
